# Survival rates and risk factors for dental prostheses in patients with maxillofacial defects: a retrospective cohort study

**DOI:** 10.3389/froh.2026.1858079

**Published:** 2026-05-28

**Authors:** Menghan Miao, Mariko Hattori, Jun Aida, Noriyuki Wakabayashi

**Affiliations:** 1 Department of Advanced Prosthodontics, Graduate School of Medical and Dental Sciences, Institute of Science Tokyo, Tokyo, Japan; 2 Department of Dental Public Health, Graduate School of Medical and Dental Sciences, Institute of Science Tokyo, Tokyo, Japan

**Keywords:** denture failure, maxillofacial abnormalities, prosthesis failure, risk factors, survival analysis

## Abstract

**Purpose:**

This retrospective cohort study evaluated survival rates of removable prostheses in patients with maxillofacial defects and assessed risk factors for prosthesis fracture, to provide descriptive data to aid clinicians in patient counseling and treatment planning.

**Materials and methods:**

Patients who initially visited the Maxillofacial Prosthetics Clinic at Tokyo Medical and Dental University Hospital in 2015 and received prostheses were included. Variables collected included prosthesis delivery date, arch treated, prosthesis type, opposing arch status, fracture occurrence, and denture base material. Prosthesis survival was estimated using the Kaplan–Meier method. Potential risk factors were analyzed by Cox proportional hazards regression.

**Results:**

Among 302 prostheses, 111 fractured over a median follow-up of 1.6 years. The 1- and 5-year prosthesis survival rates were 85.1% and 47.7%, respectively. Opposing arch status was the only significant risk factor for fracture. Relative to complete dentures, implant-supported prostheses, teeth with removable partial dentures, and natural teeth showed 3.026, 2.395, and 2.794-fold higher hazards of fracture, respectively.

**Conclusions:**

This research reveals survival rates of removable prostheses in patients with maxillofacial defects and highlights opposing arch status as a significant fracture risk factor. These findings emphasize the need to consider opposing arch conditions during treatment planning and patient counseling on prosthesis longevity.

## Introduction

Patients with maxillofacial defects often require prosthetic treatment to restore both esthetics and function. The longevity of removable prostheses worn by these patients is crucial, as they significantly improve patients’ quality of life and social interactions by restoring defects resulting from congenital conditions, developmental disorders, trauma, or oncological surgeries (1). Maxillofacial prostheses play a vital role in this rehabilitation process. Despite the patient satisfaction reported in terms of esthetics, phonetics, and functionality, the longevity of these prostheses can be compromised by various factors, including biological, mechanical, and maintenance-related issues, leading not only to fractures but also to wear, loss of retention, functional deterioration, and the need for repair or replacement (2–4).

The success of prosthodontic treatments is heavily dependent on time-related factors (5). Numerous studies have explored the survival of conventional prostheses, but comprehensive data on prostheses worn by patients with maxillofacial defects remain limited. Factors influencing complications include biological aspects (e.g., sex and health status), as well as technical elements (e.g., framework design, occlusal patterns, type of prosthesis, material used, and the condition of the opposing arch) (6–9). Understanding the prevalence and nature of complications associated with these prostheses is critical for improving clinical outcomes.

Occlusal forces exerted during mastication and other oral functions are a primary source of mechanical stress on prostheses. Previous studies have demonstrated that the bite strength and masticatory force of natural dentition are approximately four to five times greater than those of artificial teeth in complete dentures (10). Implant-supported prostheses create stress concentration points on the maxillofacial prosthesis due to the rigid connection between the implant and surrounding bone tissue (11). In cases where natural teeth oppose removable partial dentures (RPDs), the differing mobility of natural teeth and the relatively insufficient retention and stability of the dentures might contribute to uneven occlusal force distribution (12). Additionally, more than 90% of patients wear dentures intermittently (daytime or nighttime only), which compromises occlusion for the opposing prosthesis and results in occlusal forces concentrating on areas containing natural dentition; when dentures are worn, they help distribute these forces more evenly across a larger area (13–15). Patients wearing complete dentures experience lower occlusal forces. Their balanced occlusion designs stabilize the dentures during oral functions, minimize movement, and distribute force evenly to the opposing arch and supporting tissues (10, 16). Selecting appropriate materials and designs requires solid evidence, and it is important for patients to be informed and involved in the decision-making process. Ahmed et al. (17) reported that metal-based prostheses, by virtue of their higher strength and excellent fatigue-resistance performance, tend to exhibit a longer median survival time in comparison to acrylic resin prostheses. The number of abutment teeth (18, 19), posterior occlusal support (8), endodontic status and periodontal status (20) have been previously reported to impact prosthesis survival. In head and neck cancer patients, prosthesis longevity may also be influenced by changes in prosthesis use after radiotherapy (21), psychosocial aspects such as mental health (22, 23), and altered masticatory function after surgical intervention (24).

This research is based on a larger cohort of patients with maxillofacial defects, allowing for a more robust evaluation of long-term survival rates and risk factors associated with prosthesis survival. Our study focuses on patients with maxillofacial defects, offering insights not previously addressed in the literature. Therefore, this study aims to evaluate the survival rates of prostheses and identify key risk factors associated with their fractures in patients treated at the Maxillofacial Prosthetics Clinic of Tokyo Medical and Dental University Hospital, seeking to offer insights that can guide clinical decision-making and improve long-term outcomes. As the null hypothesis, it was assumed that prosthesis survival would not be significantly affected by any of the examined risk factors.

## Materials and methods

This study was designed as a retrospective cohort study. Using the hospital's patient record system, patients who initially visited the Maxillofacial Prosthetics Clinic at Tokyo Medical and Dental University Hospital between January 1 and December 31, 2015 were identified (*n* = 255). Patients were included if they had been provided with clasp-retained RPDs, complete dentures, and/or maxillofacial prostheses, and were excluded if their dentures were immediate RPDs (e.g., surgical obturator or spacer), palatal augmentation prosthesis, palatal lift prosthesis or prosthesis for cleft lip and/or palate. In addition, patients who had not visited the hospital for dental recall by the end of the study period were not included in the study. Data were gathered from the dental records. Of the 255 patients initially identified, 108 were excluded after applying the inclusion and exclusion criteria. A total of 147 patients with 302 prostheses were included in the final analysis. This study was approved by the ethics committee at Tokyo Medical and Dental University (D2016–085). In view of the retrospective nature of the study and the fact that all data were fully anonymized, the requirement for informed consent was waived.

Variables collected were sex (male, female), age (<69 or ≥69 years), date of delivery of the prosthesis, date of the last consultation, arch treated (maxilla, mandible), type of prosthesis (dental prosthesis, maxillofacial prosthesis), opposing arch status (implant-supported prosthesis, teeth and RPD, complete denture, natural teeth), status of the prostheses (fractured or not) and material of the denture base (metal, acrylic). Clasp-retained RPDs and complete dentures were classified as normal prostheses.

In this study, survival time was defined as the period from the date of prosthesis delivery to the date of documented fracture or last follow-up. Prostheses with no recorded fracture event during the observation period were considered to have survived. A life-table analysis was performed to assess the survival rates of the prostheses. Kaplan–Meier survival analysis was performed to show the survival curve. The log-rank test was used for single factor analysis to compare survival between different groups. Prosthesis survival rates at 1 and 5 years were calculated, and differences in survival rates based on the opposing arch status were evaluated. Cox’s proportional hazard regression analysis was used to test bivariate and multivariate associations between each variable and prosthesis survival time. Potential confounders, including age, sex, arch treated, prosthesis type, and denture base material, were included in the multivariable model to adjust for their effects. *P*-values < 0.05 were considered to be statistically significant.

Data were analyzed using the statistical software program STATA SE (ver. 14.1, Stata Corp., College Station, TX, USA).

## Results

Demographic and clinical characteristics of the sample are shown in Table 1. Of the 302 prostheses included in this study, 169 were placed in men and 133 in women. The patients had a median (interquartile range [IQR]) age of 69 (17) years at the date of prosthesis placement and were followed up for a median (IQR) of 1.6 (2.2) years after delivery of the prosthesis.

**Table 1:** Description of the demographic and clinical data of the study sample.

Variable		Data
Patients, n		302
Sex, *n* (%)	Male	169 (56.0)
	Female	133 (44.0)
Age (years), median (IQR)		69 (17)
Observation period (years), median (IQR)		1.6 (2.2)
Arch treated, *n* (%)	Maxilla	143 (47.4)
	Mandible	159 (52.6)
Type, *n* (%)	Normal prosthesis	99 (32.8)
	Maxillofacial prosthesis	203 (67.2)
Status of opposing arch, *n* (%)	Implant-supported prosthesis	12 (4.0)
	Teeth and RPD	127 (42.1)
	Complete denture	59 (19.5)
	Natural teeth	104 (34.4)
Material of denture base, *n* (%)	Acrylic	281 (93.0)
	Metal	21 (7.0)

IQR, interquartile range; RPD, removable partial denture.

A life table is shown in Table 2. During the observation period, 111 (36.8%) prostheses experienced fractures. The general survival rate at 1 year was 85.1% and at 5 years was 47.7%.

**Table 2:** Life table showing the cumulative survival rate of prosthesis (*n* = 302).

Interval (years)	Prosthesis failure	Survival rate	Standard error	95% CI
0	0	1		
1	41	0.8508	0.0216	0.8026–0.8880
2	35	0.6871	0.0305	0.6229–0.7427
3	18	0.5687	0.0360	0.4951–0.6357
4	6	0.5187	0.0383	0.4413–0.5906
5	3	0.4774	0.0420	0.3931–0.5568
6	5	0.3887	0.0495	0.2920–0.4841
7	2	0.3455	0.0526	0.2448–0.4482
8	1	0.3023	0.0613	0.1888–0.4239

CI, confidence interval.

**Figure 1:**
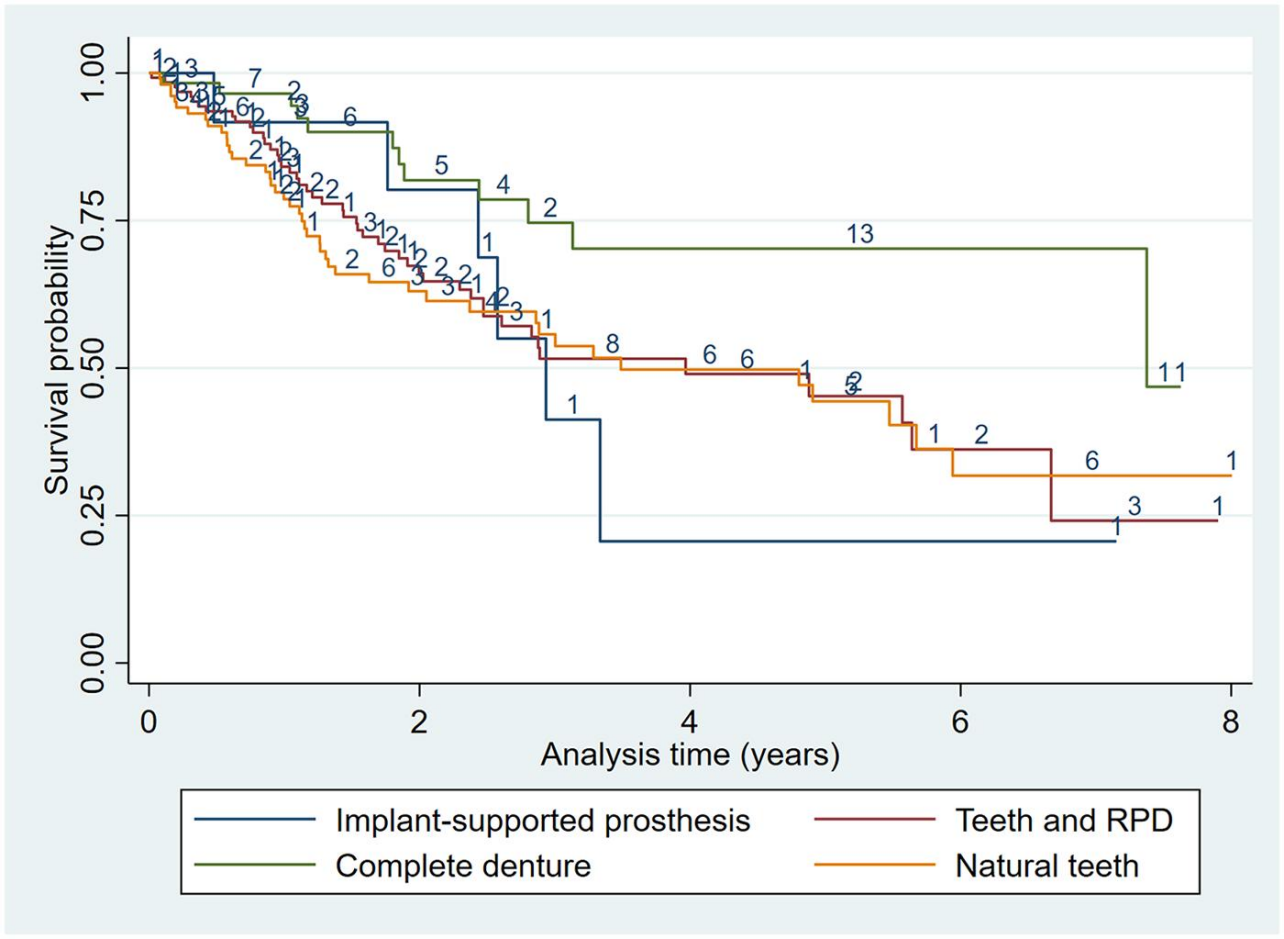
Kaplan–meier survival curves for opposing arch status (*n* = 302). RPD, removable partial denture.

Single factor analysis using the log-rank test showed that no variable had a significant influence on prosthesis survival (Table 3). The Kaplan–Meier survival curve for the opposing arch status is shown in Figure 1. The 5-year survival rates for implant-supported prosthesis, teeth and RPD, complete denture, and natural teeth were 20.6%, 45.2%, 70.2% and 44.3%, respectively.

**Table 3:** Single factor analysis of prosthesis fractures by log-rank test (*n* = 302).

Variable	*P*-value
Age	0.860
Sex	0.592
Arch treated	0.218
Type of prosthesis	0.416
Denture base material	0.297
Opposing arch status	0.053

Table 4 shows the incidence of denture fracture and the hazard ratios estimated using Cox regression. Opposing arch status was the only significant factor in both univariable and multivariable analyses. In the univariable model, hazard ratios (HRs) of denture fracture for opposing arch status relative to complete dentures were as follows: teeth and RPD (HR, 2.193; 95% CI, 1.166–4.126; *P* = 0.009) and natural teeth (HR, 2.352; 95% CI, 1.241–4.458; *P* = 0.015). In the multivariable model, relative to complete dentures, implant-supported prostheses (HR, 3.026; 95% CI: 1.075–8.518; *P* = 0.036), teeth and RPD (HR, 2.395; 95% CI: 1.255–4.572; *P* = 0.008), and natural teeth (HR, 2.794; 95% CI: 1.356–5.759; *P* = 0.005) showed significant associations with denture fracture.

**Table 4:** Incidence of denture fracture and univariable and multivariable hazard ratios of denture fracture by Cox regression (*n* = 302).

Variable		n (%)	Incidence of denture fracture (%)	Univariable		Multivariable	
				HR	95% CI	HR	95% CI
Age	<69 years	151 (50.0)	37.1	Ref.		Ref.	
	≥69 years	151 (50.0)	36.4	0.967	(0.666–1.403)	1.236	(0.819–1.866)
Sex	Male	169 (56.0)	37.3	Ref.		Ref.	
	Female	133 (44.0)	36.1	0.903	(0.620–1.314)	0.947	(0.646–1.389)
Arch treated	Maxilla	143 (47.4)	32.2	Ref.		Ref.	
	Mandible	159 (52.6)	40.9	0.789	(0.540–1.152)	0.747	(0.502–1.110)
Type of prosthesis	Normal RPD	99 (32.8)	31.3	Ref.		Ref.	
	Maxillofacial prosthesis	203 (67.2)	39.4	1.188	(0.784–1.800)	0.973	(0.613–1.544)
Denture base material	Metal	21 (7.0)	19.0	Ref.		Ref.	
	Acrylic	281 (93.0)	38.1	1.69	(0.922–4.589)	1.711	(0.620–4.726)
Opposing arch status	Complete denture	59 (19.5)	20.3	Ref.		Ref.	
	Implant-supported prosthesis	12 (4.0)	50.0	2.297	(0.861–6.126)	3.026	(1.075–8.518)*
	Teeth and RPD	127 (42.1)	38.6	2.193	(1.166–4.126)*	2.395	(1.255–4.572)**
	Natural teeth	104 (34.4)	42.3	2.352	(1.241–4.458)**	2.794	(1.356–5.759)**

CI, confidence interval; HR, hazard ratio; Ref, reference; RPD, removable partial denture.

* *P* < 0.05.

** *P* < 0.01.

## Discussion

The null hypothesis of the present study was rejected, as opposing arch status was found to significantly affect prosthesis survival. This study retrospectively analyzed a cohort of 147 patients who received oral rehabilitation with prostheses manufactured in a single university hospital clinic. Focus was placed on factors affecting the survival of removable prostheses in patients with maxillofacial defects, yielding important clinical information. The study sample was diverse, including prostheses fabricated from different materials (e.g., acrylic and metal) and incorporating various designs (e.g., clasp-retained and implant-supported maxillofacial prostheses). It also encompassed a wide spectrum of clinical situations, ranging from patients with congenital maxillofacial defects to those who had experienced post-traumatic or post-oncological surgical deficits. This broad sampling approach was intended to capture the complexity and variability inherent in prosthetic treatments for patients with jaw defects. Regarding long-term survival, the 1-year and 5-year prosthesis survival rates were 85.1% and 47.7%, respectively. This indicates significant durability challenges and highlights the difficulties faced in sustaining the effectiveness of prostheses over time. These findings indicate that prosthetic treatments in patients with maxillofacial defects face certain challenges during long-term use and are prone to problems such as fracture, which affect their service life. Despite previous studies reporting high survival rates and patient satisfaction in terms of esthetics, phonetics, and functionality of prostheses of patients with maxillofacial defects, this study reveals the limitations in their durability.

Among the various factors that were analyzed, the opposing arch status was identified as a significant risk factor for prosthesis fractures. Relative to complete dentures, the three types of opposing arch status (i.e., implant-supported prostheses, natural teeth with RPDs, and natural dentition) all significantly reduced prosthesis survival time. The stronger occlusal forces exerted by natural dentition may contribute to an increased risk of prosthesis fracture and stress concentration points caused by the rigid bone-implant interface in implant-supported prostheses might also increase this risk. The uneven distribution of occlusal load due to the differential mobility and limited stability of RPDs may be additional causes (10–12). In contrast, prostheses opposing complete dentures exhibited longer survival, likely due to their lower occlusal forces and the stabilizing effects of balanced occlusion, which helps evenly distribute masticatory loads and minimize stress concentration (10, 16). This finding has important clinical implications: treatment planning should include careful evaluation of opposing arch conditions to guide the selection of appropriate removable prostheses.

Regarding the base materials of the prostheses, the observed trend of longer survival in metal-based prostheses aligns with previous studies (17). However, no statistically significant difference in survival rates was observed between the metal and acrylic groups (95% CI: 0.922–4.589; *P* = 0.304), possibly due to sample size limitations. The lack of statistical significance does not imply that no difference existed; rather, the observed differences were insufficient to support a definitive conclusion within the limits of this study design. Continuous investigation is needed to clarify the effect of prosthesis base materials in patients with maxillofacial defects. In addition, emerging high-performance polymers such as polyetheretherketone and polyetherketoneketone, which combine rigidity with controlled flexibility, are considered promising alternative materials for prosthetic frameworks and warrant further investigation in this patient population (25).

Sex was not a statistically significant factor in this study, which is consistent with Nisser et al. (18) and Tada et al. (20) reporting no association between sex and RPD survival. However, in the study by Nisser et al. (18), sex was identified as a significant risk factor for abutment failure in RPDs (95% CI: 0.322–0.912; *p* = 0.021). Previous studies have shown that males generally exert higher masticatory forces than females, potentially increasing mechanical stress on prostheses and fracture risk (26). Although not significant here, the interaction between sex and occlusal conditions may still be clinically relevant and warrants further investigation.

The impact of the condition of the abutment teeth on prosthesis survival was previously investigated. Wöstmann et al. (19) reported that the prosthesis survival rate increased with the number of abutment teeth, from 70.9% with one abutment to 97.9% with four. Similarly, a retrospective study (18) found that the main cause of prosthesis failure was the loss of abutment teeth (40.6%). These reports suggest that the condition of the abutment teeth plays a key role, which may also apply to our cases. Koichi et al. (8) showed a lower replacement risk in patients with better posterior support (Eichner index A-B3 relative to B4-C), supporting the idea that occlusal support affects survival. Tada et al. (20) identified crown-root ratio and pocket depth as significant predictors, highlighting the role of periodontal factors. In addition, reduced denture use among head and neck cancer patients after radiotherapy (21) and decreased bite force after jaw resection (24) may contribute to a shorter prosthesis lifespan, especially in this population. These factors can also impact the denture lifespan.

This study had several limitations. Like any retrospective study, the design and reliance on existing patient records for data collection introduce potential biases. Additionally, the study was conducted in a university setting, which may not fully represent the diversity of clinical practice. The number and experience of operators involved in designing and delivering the prostheses are also variables that need to be considered when interpreting and generalizing the findings. Moreover, several factors known to impact prosthesis lifespan were not included in the present analysis. Potential sources of bias were also considered, including selection bias due to the single-center design, information bias arising from retrospective data collection, and loss to follow-up resulting from exclusion of non-returning patients. In addition, although major clinical variables were adjusted for in the multivariable analysis, some potential confounders (such as prosthesis design complexity, oral hygiene status, and operator-related factors) were not available in the dataset and therefore could not be included. Future research should take these factors into account when evaluating the long-term outcomes of prosthetic treatment.

The results provide estimated survival times across denture types under different conditions, informing clinical decisions, supporting the optimization of denture selection and treatment strategies, and thereby improving communication between clinicians and patients. The study also suggests that careful consideration of denture type and comprehensive restoration is important for maximizing RPD longevity. These findings may prompt revisions in the informed consent process and warranty coverage, ensuring patients are fully informed about potential risks. Further research with larger sample sizes and longer follow-up periods is needed to confirm the results and explore additional factors impacting the survival of prostheses in patients with maxillofacial defects.

The survival rates of prostheses worn by patients with maxillofacial defects showed that the opposing arch status was a significant risk factor for prosthesis fracture and suggested that it is important to consider the opposing arch status during treatment planning for patients requiring maxillofacial prostheses.

## Conclusion

Opposing arch status was identified as a significant risk factor for prosthesis fracture in patients with maxillofacial defects. This factor should be carefully considered during treatment planning for removable prostheses.

## Data Availability

The original contributions presented in the study are included in the article/Supplementary Material, further inquiries can be directed to the corresponding author.
